# H3N2 canine influenza virus causes severe morbidity in dogs with induction of genes related to inflammation and apoptosis

**DOI:** 10.1186/1297-9716-44-92

**Published:** 2013-10-03

**Authors:** Young Myong Kang, Heui Man Kim, Keun Bon Ku, Eun Hye Park, Jung Yum, Sang Heui Seo

**Affiliations:** 1 Laboratory of Influenza Research, College of Veterinary Medicine, Chungnam National University, Daejeon, South Korea; 2 Institute for Influenza Virus, College of Veterinary Medicine, Chungnam National University, Daejeon, South Korea

## Abstract

Dogs are companion animals that live in close proximity with humans. Canine H3N2 influenza virus has been isolated from pet dogs that showed severe respiratory signs and other clinical symptoms such as fever, reduced body weight, and interstitial pneumonia. The canine H3N2 influenza virus can be highly transmissible among dogs via aerosols. When we analyzed global gene expression in the lungs of infected dogs, the genes associated with the immune response and cell death were greatly elevated. Taken together, our results suggest that canine H3N2 influenza virus can be easily transmitted among dogs, and that severe pneumonia in the infected dogs may be partially due to the elevated expression of genes related to inflammation and apoptosis.

## Introduction

The influenza virus infecting vertebrates is categorized as genera A, B, and C on the basis of the antigenic differences in their nucleoprotein (NP) and matrix proteins (M) [1,2]. Influenza A virus is further categorized according to the 16 subtypes of hemagglutinin (HA) and 9 subtypes of neuraminidase (NA) [2,3]. All the known influenza A viruses can be found in aquatic birds, and have a broad spectrum of hosts, including chickens, cats, dogs, pigs, whales, and humans [2]. The transmission of influenza from animals to humans does not give rise systemically to a pandemic. It is predicted that pandemics caused by influenza A virus may occur 3–4 times per century. During the 20^th^ century, humans experienced three pandemics: an H1N1 pandemic in 1918, an H2N2 pandemic in 1957, and an H3N2 pandemic in 1968 [4-9]. The first pandemic of the 21st century occurred in 2009, caused by the swine-originated H1N1 influenza virus [10].

Dogs are one of the most popular companion animals and live in close contact with humans. Dogs infected with influenza A viruses, including H3N8, H3N2, and H5N1, have been previously reported [11-13]. An H3N8 influenza A virus was isolated in January 2004 from the lung tissues of racing greyhound dogs that suffered from severe clinical signs such as high fever, hemorrhagic tracheitis, bronchopneumonia, and vasculitis [11,14]. Further cases of H3N8 infections in racing dogs were reported in 6 US states in the summer of 2004, and in 11 US states in 2005 and 2006 [11,14,15]. More H3N8 influenza viruses were isolated from archived tissues of greyhound dogs that died in Florida in late 2003, from greyhound dogs in Texas in 2004, and from 2 pet dogs in 2005 [14]. Fatally infected dogs that had fed on the carcasses of chickens infected with highly pathogenic (HP) H5N1 influenza virus were reported in Thailand [12]. Serological study of 629 village dogs for H5 antibodies in central Thailand showed that some of those dogs were positive for H5N1 infection [16]. These studies were conducted to evaluate dogs as potential vectors in the transmission of HP H5N1 influenza virus to humans [17,18]. The studies showed that dogs inoculated with the HP H5N1 influenza virus did not show severe clinical signs, but suffered only from conjunctivitis and showed transiently elevated body temperature. All inoculated dogs were seroconverted, and transmission of viruses from inoculated dogs to contact dogs was not observed. An outbreak of avian origin H3N2 influenza virus in pet dogs occurred in South Korea in 2007 [13]. When beagles were infected with the canine H3N2 influenza virus, they suffered from high fever, necrotizing tracheobronchitis, and bronchioalveolitis [13]. Avian origin H3N2 influenza virus was also isolated from clinically ill dogs in southern China in 2006 and 2007 [19].

In this study, we are interested in finding out the pathogenicity of a recently isolated canine H3N2 influenza virus in dogs. Therefore, we examined the clinical signs of dogs infected with a canine H3N2 influenza virus and the morphology of a recently isolated, avian origin, canine H3N2 influenza virus. We used broad-spectrum microarray analysis and real-time PCR to investigate the possible pathogenesis by which the H3N2 influenza virus infected the dogs.

## Materials and methods

### Virus

A canine influenza virus, A/Korea/S1/Canine/2012 (H3N2), was isolated from a dog showing respiratory clinical signs in an animal clinic in Korea in 2012 using a nasal swab in PBS (pH 7.4) to inoculate a 10-day-old hen egg.

### Animals

Approximately 2-month-old Korean farm dogs (Korean mongrel, *n* = 3) were serologically tested using a hemagglutination-inhibition (HI) assay with 0.5% turkey red blood cells. They were negative for human influenza viruses (H1N1, H3N2, and Influenza virus B) and canine H3N2 influenza virus. All animal experiments were performed at a biosafety level 3 (BSL-3) facility approved by the Korean government.

### Ethics statement

This study was carried out in strict accordance with the recommendations in the Guide for the Care and Use of Laboratory animals of Korean veterinary quarantine and service. The animal experiments were approved by the Animal Experimental Ethics Committee at the Chungnam National University.

### Canine H3N2 influenza virus imaging by transmission electron microscopy

The H3N2 canine influenza virus was propagated in a 10-day-old hen at 35 °C for 60 h. The allantoic fluid containing the virus was harvested and concentrated to one-tenth of the original volume using an Amicon concentrator apparatus. The concentrated viruses were purified using a 20–75% sucrose continuous gradient at 26 000 rpm (4 °C) for 2 h. The purified viruses were fixed in a 2.5% paraformaldehyde-glutaraldehyde mixture buffered with 0.1 M phosphate (pH 7.2) for 2 h, postfixed in 1% osmium tetroxide in the same buffer for 1 h, dehydrated in graded ethanol and propylene oxide, and embedded in Epon-812. The sample was stained with uranyl acetate and lead citrate, and examined under a CM 20 (Philips, Netherlands) electron microscope.

### Measurement of clinical signs and viral titers in infected dogs

The dogs (*n* = 3) were infected intranasally (i.n.) with 10^6^ EID_50_ of canine H3N2 influenza virus, and the rectal body temperature and body weight were measured for 14 days after infection. Daily swabs were obtained from the trachea and rectum, suspended in PBS (pH 7.4), and used to inoculate a 10-day-old hen egg to determine the log_10_ egg infectious dose 50/mL (log_10_ EID_50_/mL). The EID_50_/mL was calculated as previously described [20]. Three other Korean mongrels were mock-infected with PBS as controls.

### Clinical scores of infected dogs

Clinical signs were observed in the dogs for 14 days after infection. The clinical scores were the mean score of activity and respiratory signs. Ocular and nasal discharges were scored as follows: 0 for no discharge, 1.0 for serous discharge, 2.0 for mild mucus, and 3.0 for copious mucus. Cough was scored as 0 for no cough, 1.0 for mild cough, 2.0 for moderate or persistent cough, and 3.0 for severe cough accompanied by choking or retching sounds. Their appetite was scored as 0 for present, 1.0 for mild anorexia, 2.0 for mild anorexia and adipsia, and 3.0 for persistent anorexia and depression. Their hair was scored as 0 for normal, 1.0 for mildly rough, 2.0 for moderately rough, and 3.0 for severely rough hair.

### Aerosol transmission of canine H3N2 influenza virus in dogs

Aerosol transmission of canine H3N2 influenza virus was performed as described in reference [21]. The 3 experimental dogs infected with 10^6^ p.f.u. of canine H3N2 influenza virus were placed in a cage, and 1 day later, 3 naïve dogs in the cage were placed about 90.0 cm apart from the experimental dogs. The nasal and cloacal swabs in PBS (pH 7.4) were acquired 3 and 5 days after the transmission setup. The viral titers in swabs were determined by EID_50_/mL.

### Imaging of gross lung lesions

The experimentally-infected dogs were euthanized at day 5 post infection using intravenous injection of T61 (0.3 mL/kg of body weight; Intervet, USA). Lung tissues (left cranial lobes) were then collected. The lung tissues of the control dogs were also acquired in a similar manner. Images of the ventral and dorsal lesions of lungs were captured.

### Histopathological staining and immunohistochemistry of lung tissues

The lung tissues were fixed by immersing in 10% neutral buffered formalin, and were embedded in paraffin. Five-micrometer-thick sections were made from the paraffin-embedded tissues, and were stained with hematoxylin and eosin (H&E) as described previously [22]. The stained tissues were evaluated under an Olympus DP70 microscope (Olympus Corporation, Tokyo, Japan).

Five-micrometer-thick sections were stained with mouse anti-influenza A virus nucleoprotein antibody (Serotech, United Kingdom) [23]. The tissue sections were deparaffinized and hydrated in distilled water, followed by fixing with 100% chilled acetone for 2 h for permeabilization. The endogenous peroxidase activity was blocked by incubating the sections in 3% H_2_O_2_ for 15 min at 37 °C before the sections were blocked with 5% bovine serum albumin in PBS (pH 7.4) for 1 h. The blocked tissue sections were labeled with mouse anti-influenza A virus nucleoprotein antibody (1:1000 dilution) by incubating at room temperature for 1 h. The labeled tissue sections were stained with biotin-labeled goat anti-mouse immunoglobulin (Vector, USA), VECTASTAIN ABC-AP (Vector, USA), and Vector red alkaline phosphatase substrate (Vector, USA). The stained tissue sections were counterstained with hematoxylin QS (Vector Laboratories, Burlingame, CA, USA), after which the stained sections were evaluated under an Olympus DP70 microscope (Olympus Corporation, Tokyo, Japan).

### Gene expression and transcription analysis using DNA microarray in the dog lung tissues

Sterile mortars and pestles were used to pulverize about 1 g of each lung tissue sample in liquid nitrogen; the samples were crushed until a fine powder remained. The powder from each sample was placed into a cold 2-mL microtube, and 1000 μL of TRIzol (Invitrogen, USA) was added and mixed by inverting the tube to help in cell lysis. The TRIzol solution containing the disrupted tissue was then centrifuged at 12 000 *g* for 10 min at 4 °C. The colorless supernatant phase was collected into a new 2-mL tube and incubated in ice for 5 min, after which 200 μL of chloroform (Sigma-Aldrich, USA) was added to the samples, vigorously shaken by hand, and incubated again in ice for 2 min. This solution was centrifuged at 12 000 *g* for 15 min at 4 °C, and the aqueous phase was collected into a new cold tube. Chilled isopropyl alcohol (500 μL; Sigma-Aldrich, USA) was added to the solution for RNA separation, mixed by inverting the tube, and incubated in ice for 10 min. The solution was centrifuged at 12 000 *g* for 10 min at 4 °C, after which the supernatant was decanted, and the pellet, recovered. Then, the RNA pellet was washed with 1000 μL of 75% chilled ethanol (Sigma-Aldrich, USA), and the tube was inverted to wash the RNA. The solution was centrifuged at 9000 *g* for 5 min at 4 °C. The supernatant was then decanted, the pellet was recovered, and was air-dried at room temperature for approximately 10–15 min. RNA was dissolved into 50 μL of DEPC-treated water by pipetting the solution a few times and treated with DNase (Qiagen, USA).

The synthesis of target cRNA probes and hybridization were performed on equal amounts of control and test RNA from each dog using Agilent’s Low RNA Input Linear Amplification kit (Agilent Technology, USA) according to the manufacturer’s instructions. For each sample, 1 g of total RNA and T7 promoter primer mix was incubated at 65 °C for 10 min. A cDNA master mix (5× First strand buffer, 0.1 M DTT, 10 mM dNTP mix, RNase-Out, and MMLV-RT) was prepared and added to the reaction mix. The samples were incubated at 40 °C for 2 h and then at room temperature; the dsDNA synthesis was terminated by incubating at 65 °C for 15 min. The transcription master mix was prepared according to the manufacturer’s protocol (4× Transcription buffer, 0.1 M DTT, NTP mix, 50% PEG, RNase-Out, inorganic pyrophosphatase, T7-RNA polymerase, and Cyanine 3-CTP). Transcription of the dsDNA was achieved by adding the transcription master mix to the dsDNA reaction samples, and incubating at 40 °C for 2 h. Amplified and labeled cRNA was purified on a cRNA Cleanup Module (Agilent Technology) according to the manufacturer’s protocol. Labeled cRNA target was quantified using an ND-1000 spectrophotometer (NanoDrop Technologies, Inc., Wilmington, DE, USA). After checking labeling efficiency, fragmentation of cRNA was performed by adding 10× blocking agent and 25× fragmentation buffer, and incubating at 60 °C for 30 min. The fragmented cRNA was resuspended in 2× hybridization buffer and directly pipetted onto an assembled Agilent Canine Oligo Microarray (44 K) [24]. The arrays were hybridized at 65 °C for 17 h using an Agilent Hybridization oven (Agilent Technology, USA). The hybridized microarrays were washed according to the manufacturer’s washing protocol (Agilent Technology, USA).

The hybridized images were scanned using Agilent’s DNA microarray scanner and quantified with Feature Extraction Software (Agilent Technology, Palo Alto, CA, USA). All data normalization and selection of fold changes in gene expression were carried out using GeneSpringGX 7.3 (Agilent Technology, USA). The averages of normalized ratios were calculated by dividing the average of normalized signal channel intensity by the average of normalized control channel intensity. Functional annotation of genes was performed according to the Gene Ontology database [25], BioCarta [26], GenMAP [27], DAVID bioinformatics resource [28], and National center for biotechnology information [29]. GEO accession number is GSE44545.

### Quantification of dogs’ genes by real-time polymerase chain reaction (PCR)

Total RNA was collected from lung tissues (1 g) of dogs (*n* = 3 per group) that were infected with canine H3N2 influenza virus using TRIzol reagent (Invitrogen, Carlsbad, CA, USA). One milliliter of TRIzol reagent (Invitrogen) was added to tubes containing tissues and incubated at room temperature for 5 min. Chloroform (200 L) was added and the solution was mixed by vortexing for 15 s and centrifuged for 15 min (12 000 rpm, 4 °C). The upper RNA-containing band was collected and mixed with 500 L of isopropanol (Sigma-Aldrich, St. Louis, MO, USA) in a new 1.5 mL tube. Each sample was centrifuged for 10 min (10 000 rpm, 4 °C), and the RNA-containing pellet was washed with 100 μL of 75% ethanol in water by centrifuging for 5 min (10 000 rpm and 4 °C). The washed RNA was resuspended in 50 μL of diethyl pyrocarbonate (DEPC)-treated water.

The mRNA of dogs’ inflammatory cytokines and chemokines were quantified using quantitative real-time PCR. To synthesize the cDNA, 1 μL of oligo dT primers (0.5 pmoles) (Promega, Madison, WI, USA) was added to a total volume of 9 μL in a 0.05 mL tube. The mixture was reacted for 5 min at 70 °C prior to incubation for 5 min at 4 °C. Then, each sample received 4 μL of 25 mM MgCl_2_, 4 μL of 5X reverse transcriptase enzyme buffer, 1 μL of RNase inhibitor, 1 μL of reverse transcriptase, and 1 μL of dNTP (10 mM). Each sample was incubated for 5 min at 25 °C, for 60 min at 42 °C, and 15 min at 70 °C. SYBR Green-based real-time PCR was performed using a Roto-Gene 6000 apparatus (Corbett, Mortlake, Australia) and SensiMix Plus SYBR (Quantace, London, UK) based on recommendations of the manufacturer. A duplicate of each sample was run. A total volume of 20 μL containing 2 μL cDNA, 10 μL SYBR mixture, and gene-specific primers (Table 1) (1 μL of forward primer (20 pmole) and 1 μL of reverse primer (20 pmole)) was used for 40 cycles of PCR: 5 s at 95 °C, 15 s at 60 °C, and 25 s at 72 °C. Cytokine and TLR expression levels in tissues were normalized to those of dog glyceraldehyde-3-phosphate dehydrogenase (GAPDH). The results of real-time PCR were quantified by the comparative threshold method after deductions of data from uninfected control dogs.

**Table 1:** Real-time PCR primer sequences

**Group**	**Gene** **GAPDH**	**Forward primer** **TCA ACG GAT TTG GCC GTA TTG G**	**Reverse primer** **TGA AGG GGT CAT TGA TGG CG**
TLR	TLR2	TGT TGT TGG GCA ACT GAA AA	TTA AAC AAG TGG GGC AAA GG
NK cell	IL15	AGT AAC CGC GAT GAA GTG CT	CTT TGC ATC CCG TTT CAG TT
	FCIGR	CCT GGT GAT GGC ACT CCT AT	GGG TTG TGG ATC CAG AGA GA
Macrophage	MARCO	CCA GGA CTT TTG GCC ATT TA	TTC TCT TCT TGG GCT TTG GA
	MMP12	GTC ACT GCT TCG GGT ACC AT	GAT GCA TCC AGT TTC CCA GT
Neutrophil	CSF2	AAC ATC CCC TTT GAC TGC TG	AAG TCC ATG CCC CCT CTA CT
	SLPI	CCT TGG ACT GTG GAA GGT GT	ACC TGC CAG GCT TCA TTC TA
NO and ROS	GCLM	GGC GCA GGT AAA ACC AAA TA	GCT TTC CTG AAG GGC TTC TT
	HSP90AA1	GTC AGT GAC GAT GAG GCT GA	GTG ATG TCG TCA GGG TTC CT
	ADM	CGA CGT CTC AGA CCT TCT CC	CCA CGA CTT ACA GCC CAT TT
	SOD2	TGT TGT TGG GCA ACT GAA AA	TTA AAC AAG TGG GGC AAA GG
IFN	IFIT3	TTC CCC ACA AGA GCA AAA TC	GCA GGA AAA AGC TGC CTA TG
	ISG20	CGA TGG AGC TTT ACC GAC TC	ATG CAT TTC CCA AAA GCA AG
	MX1	GGA GGC TCT GTC AGG AGT TG	TGA CTG ATC CCC TGT CCT TC
Cytokine and chemokine	IL15	AGT AAC CGC GAT GAA GTG CT	CTT TGC ATC CCG TTT CAG TT
	C3	CGC TAC CAG AAC CTG AGG AG	CGG ACG ACA GGA GGT ACA TT
	IL10RB	GCC TTC ACG GAA TGT GAT TT	TAG GGG CTA AGA AAC GCA GA
	IL1R2	ATG GGT GTT TCA GCC TTC AC	CTG GAC CCA CAT TCT CGT CT
	CXCL10	TGA ACC AAA GTG CTG TTC TTA TTT	ACG ATG GAC TTG CAG GAA TC
DC	TAP1	CCA TGG CTC TCA CTA GCA CA	GCA GGC TGG AGC TCA ATT AC
	MX1	GGA GGC TCT GTC AGG AGT TG	TGA CTG ATC CCC TGT CCT TC
Apoptosis	TNFSF13	GGG CGA AAC TTA GCC TCT CT	GGT TGA GGT CAA ACC CAG AA
	NME5	AAG GGC AAT TTA TGG CAC AG	CCG CTG GTT TCT CCT TAC AA
	PYCARD	CTG CAG GAG ACC TCA CAC AA	TCC TCA TTT TCC CTG GAT TG
TH1	SPP1	TTG CAG TGA TTT GCT TTT GC	CAG ACT CGT TGG AGT CGT CA
TH2	CCL5	GCT GCT TTG CCT ACA TTT CC	TCA GGT TCC AGA TGC CCT AC
	CCL7	CCC ATC CAG AAG CTG AAG AG	GCT TGG GTT TTC TTG TCC AG

### Statistical analysis

Statistical analysis was performed using the Statistical Product and Services Solutions (SPSS) package, version 10.0 (SPSS, Cary, NC, USA). Student’s *t*-test was used. A *P*-value < 0.05 was considered to be statistically significant. The data from the infected dogs were compared with those from the uninfected dogs.

## Results

### Morphology of isolated canine H3N2 influenza virus

**Figure 1:**
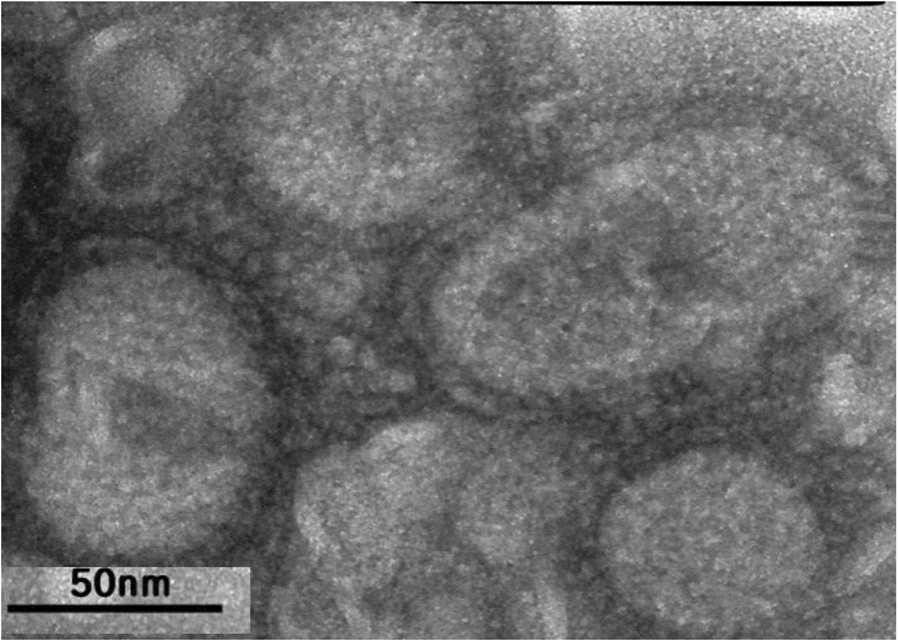
**Morphology of the canine H3N2 influenza virus.** H3N2 canine influenza virus (A/Korea/S1/Canine/2012) propagated in eggs was purified by sucrose gradient centrifugation. The purified virion was observed using a transmission electron microscope.

To determine the shape of avian origin canine H3N2 influenza virus, the purified virus was observed by transmission electron microscopy. The image shows that the virion contains an envelope and surface projections, and that the shape is pleomorphic, which is typical of enveloped viruses (Figure 1).

### Clinical signs, viral titers, and aerosol transmission of canine H3N2 influenza virus in dogs

**Figure 2:**
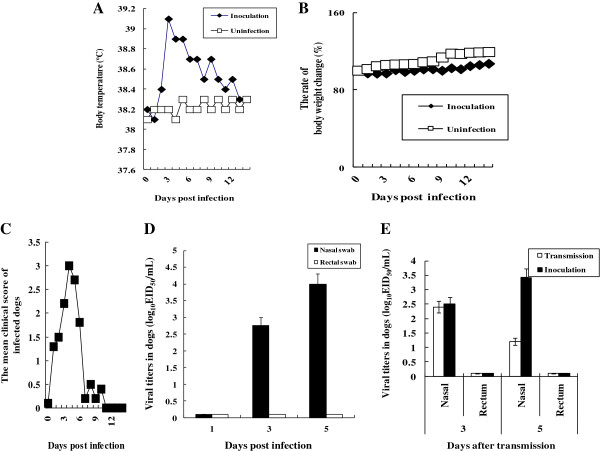
**Clinical signs and viral titer in the infected dogs, and aerosol transmission of canine H3N2 influenza virus.** Dogs (Korean mongrel, *n* = 3) were i.n. infected with A/Korea/S1/Canine/2012 (H3N2), and the rectal body temperature **(A)** and body weight **(B)** were measured for 14 days after infection. Clinical scores were measured as described in the Materials and methods **(C)**, and the viral titer **(D)** of swabs performed daily from tracheas and rectum was determined by log_10_ EID_50_/mL. To study aerosol transmission **(E)** of the canine H3N2 influenza virus, 2 dogs (Korean mongrel) infected with A/Korea/S1/Canine/2012 (H3N2) in a cage were placed 90.0 cm apart from a cage containing 3 naïve dogs 1 day after infection. The viral titer in the nasal and rectal swabs performed on 3 and 5 days after transmission setup was determined by log_10_ EID_50_/mL.

When the dogs were i.n. inoculated with H3N2, they suffered from elevated body temperature and reduced body weight, and shed the virus via the upper respiratory tract (Figure 2A-D). The rectal body temperature in the infected dogs peaked at 39.1 °C 3 days after infection (Figure 2A), and the body weight of the infected dogs decreased to 98.6% of that prior to the infection (Figure 2B). The overall clinical score peaked at 3.0 on 4 days after infection (Figure 2C). When we measured viral titers from the nasal and rectal swabs of the infected dogs, viruses were only detected in the nasal swabs with 4.0 EID_50_/mL 5 days after infection (Figure 2D).

We tested the possibility of aerosol transmission of canine H3N2 influenza virus among dogs to determine whether the avian origin canine H3N2 influenza virus is well adapted in dogs. When the cage containing the infected dogs was placed 90.0 cm apart from the cage containing naïve dogs, the viruses were detected in the nasal swabs of the naïve dogs 3 and 5 days after transmission (Figure 2E), suggesting that canine H3N2 influenza virus has an effective transmission among dogs via aerosols.

### Gross pathology, histopathology, and immunohistochemistry of the lungs of infected dogs

**Figure 3:**
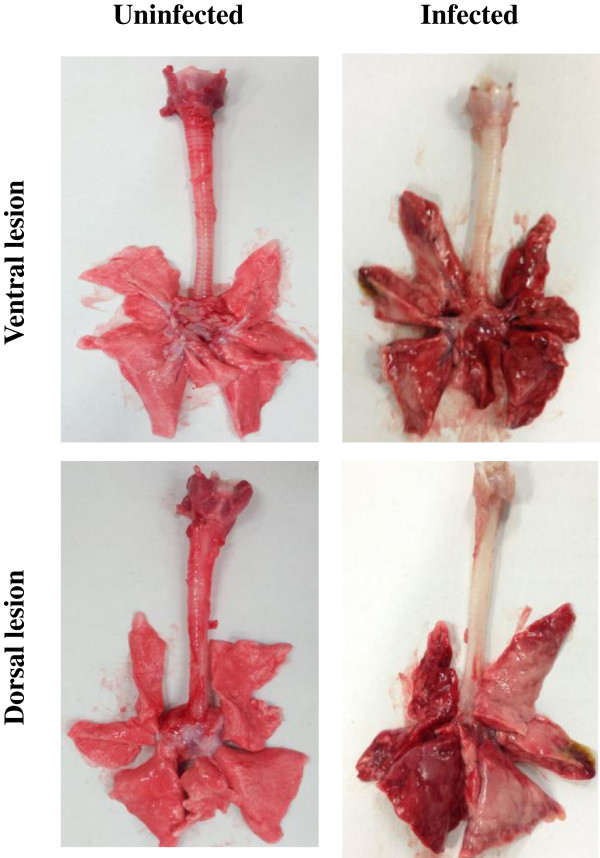
**Gross lung lesions of infected dogs.** Lungs were obtained from dogs (Korean mongrel, *n* = 2) infected with A/Korea/S1/Canine/2012 (H3N2) and from the PBS mock-infected dogs 5 days after infection. The ventral and dorsal lesions of the lungs are shown.

**Figure 4:**
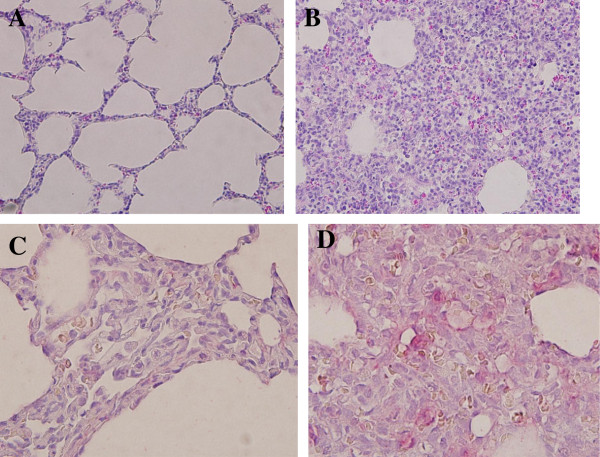
**Histopathology and immunohistochemistry of the lungs of infected dogs.** The left cranial lobes of the lungs from dogs (Korean mongrel, *n* = 2) infected with A/Korea/S1/Canine/2012 (H3N2) and from PBS mock-infected dogs were collected 5 days after infection. The lung tissues from PBS mock-infected **(A)** or infected **(B)** dogs were stained with H&E stain. In addition, the lung tissues from PBS mock-infected **(C)** or infected **(D)** dogs were stained with mouse anti-influenza A nucleoprotein antibody, biotin-labeled goat anti-mouse immunoglobulin, and red alkaline phosphatase substrate. The stained sections were imaged with an Olympus DP70 microscope.

We compared the gross pathological lesions of the lungs of the infected and uninfected dogs. The left cranial, middle, and caudal lobes of infected lungs had severe pneumonia, along with edema and hemorrhage (Figure 3). The H&E staining of the tissues showed interstitial pneumonia with infiltration of numerous inflammatory cells (Figure 4B). Lung tissues stained with the antibody against influenza A nucleoprotein were positive mainly in the pneumocytes with some alveolar macrophages (Figure 4D).

### Analysis of gene transcription induced in the lungs of infected dogs

**Figure 5:**
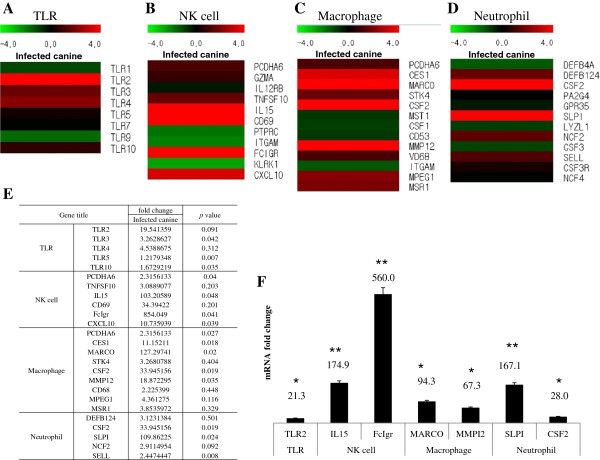
**Microarray and real-time PCR analysis of genes associated with Toll-like receptors, NK cells, macrophages, and neutrophils.** The mRNA from the left cranial lobes of the lungs from dogs (Korean mongrel, *n* = 3) infected with A/Korea/S1/Canine/2012 (H3N2) and from PBS mock-infected dogs were subjected to microarray analysis. The genes induced higher in the infected dogs were quantified by real-time PCR. **A**, Heat map of genes related to TLR; **B**, Heat map of genes related to NK cells; **C**, The fold-change comparison of genes related to NK cells; **D**, Heat map of genes related to macrophages; **E**, Microarray data of Toll-like receptors, NK cells, macrophages, and neutrophils; **F**, mRNA fold changes of selected genes of Toll-like receptors, NK cells, macrophages, and neutrophils. *P value < 0.05, ** *P* value < 0.01.

**Figure 6:**
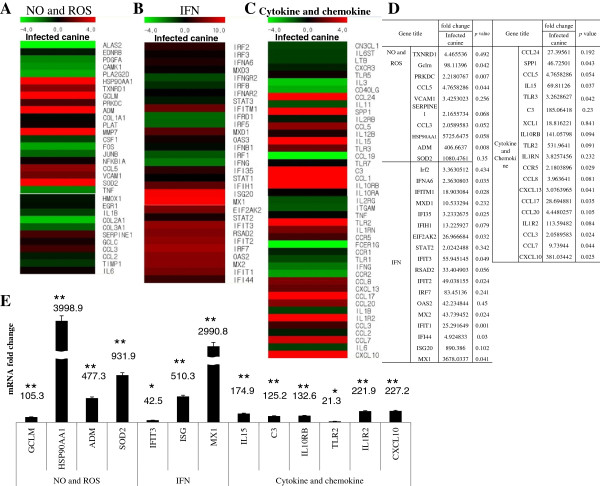
**Microarray and real-time PCR analysis of genes associated with nitric oxide and reactive oxygen species, interferon, cytokine and chemokine.** The mRNA from the left cranial lobes of the lungs from dogs (Korean mongrel, *n* = 3) infected with A/Korea/S1/Canine/2012 (H3N2) and from PBS mock-infected dogs were subjected to microarray analysis. The genes induced higher in the infected dogs were quantified by real-time PCR. **A**, Heat map of genes related to nitric oxide (NO) and reactive oxygen species (ROS); **B**, Heat map of genes related to IFN; **C**, Heat map of genes related to cytokine and chemokine; **D**, Microarray data of nitric oxide and reactive oxygen species, interferon, cytokine and chemokine; **E**, mRNA fold changes of selected genes of nitric oxide and reactive oxygen species, interferon, cytokine and chemokine. *P value < 0.05, **P value < 0.01.

We performed a microarray analysis of mRNA collected from the lungs of canine H3N2 influenza virus-infected and PBS mock-infected dogs to understand the underlying mechanism by which the virus could cause severe respiratory signs. Many genes related to innate immunity were induced, such as Toll-like receptors (TLR), natural killer (NK) cells, macrophages, neutrophils, nitric oxide (NO) molecules and reactive oxygen species (ROS), interferons (IFN), and cytokines and chemokines (Figures 5 and 6). Among TLR 1–10, TLR2 recognizes foreign substances and transmits appropriate signals to the immune cells, and it had the greatest induction (19.5-fold increase, Figure 5E). When we quantified mRNA of TLR2 by real-time PCR, the induction of TLR2 increased as much as 21.3 fold compared to the uninfected control (Figure 5F). The greatest inductions (an 854.0-fold increase) in gene expression in NK cells were also seen with that for the Fc fragment of IgG (FClGR, low affinity IIIa, receptor), which is involved in the removal of antigen-antibody complexes from the circulation (Figure 5E). When mRNA of FCIGR was quantified with real-time PCR, its induction was a 560.0-fold increase (Figure 5F). Macrophage receptor with collagenous structure (MARCO), which is involved in the innate antimicrobial immune system, had a 127.3-fold increase among macrophages (Figure 5E). When mRNA of MARCO was quantified by real-time PCR, the induction of it was 94.3-fold increase (Figure 5F). Secretory leukocyte peptidase inhibitor (SLPI), which protects epithelial tissues from serine proteases, had a 109.9-fold increase among neutrophils (Figure 5E). When mRNA of SLPI was quantified by real-time PCR the induction of it was 167.1-fold increase (Figure 5F). Among the NO- and ROS-related genes, the heat shock protein 90-kDa alpha (Hsp90AA1) involved in the proper folding of specific target proteins had a 5725.6-fold increase (Figure 6D). When mRNA of Hsp90AA1 was quantified by real-time PCR the induction of it was a 3998.9-fold increase (Figure 6E). Superoxide dismutase 2 (SOD2) transforming toxic superoxides into hydrogen peroxide and diatomic oxygen had a 1080.5-fold increase (Figure 6D). When mRNA of SOD2 was quantified by real-time PCR the induction of it was a 931.9-fold increase (Figure 6E). Adrenomedullin (ADM), which increases the tolerance of cells to oxidative stress and hypoxic injury, had a 406.7-fold increase (Figure 6D). When mRNA of ADM was quantified by real-time PCR, the induction of it was a 477.3-fold increase (Figure 6E). Among the antiviral interferon-related genes, the most highly induced were myxovirus resistance 1 (Mx1), which is involved in a specific antiviral state against influenza virus infection, and had a 3878.0-fold increase (Figure 6D). When mRNA of Mx1 was quantified by real-time PCR, the induction of it was a 2990.8-fold increase (Figure 6E). Interferon stimulated exonuclease gene (ISG20), a 20-kDa molecule, which degrades viral RNA, had an 890.0-fold increase (Figure 6D). When mRNA of ISG20 was quantified by real-time PCR, the induction of it was a 510.3-fold increase (Figure 6E). The maximum induction among the cytokine and chemokine-related genes involved in inflammation was observed in chemokine (C-X-C motif) ligand 10 (CXCL10), which is associated with chemoattraction for monocytes, macrophages, T lymphocytes, NK cells, and dendritic cells, which showed a 381.0-fold increase (Figure 6D). When mRNA of CXCL10 was quantified by real-time PCR, the induction of it was a 227.2-fold increase (Figure 6E).

**Figure 7:**
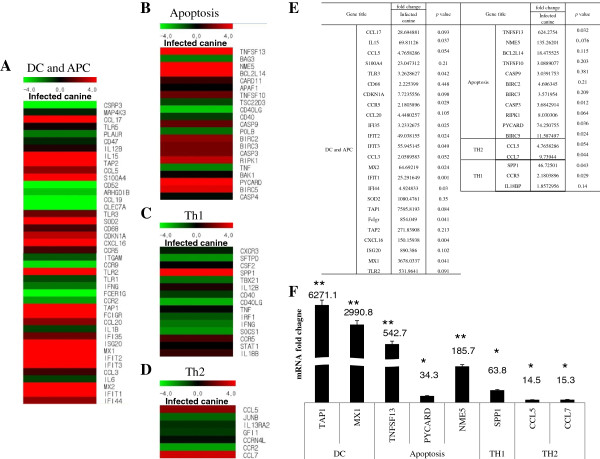
**Microarray analysis of genes associated with dendritic cells and antigen presentation, apoptosis, Th1 and Th2 CD4**^**+ **^**T lymphocytes.** The mRNA from the left cranial lobes of the lungs from dogs (Korean mongrel, *n* = 3) infected with A/Korea/S1/Canine/2012 (H3N2) and from PBS mock-infected dogs were subjected to microarray analysis. The genes induced higher in the infected dogs were quantified by real-time PCR. **A**, Heat map of genes related to dendritic cells (DC) and antigen presentation (APC); **B**, Heat map of genes related to apoptosis; **C**, Heat map of genes related to Th1 CD4^+^ T lymphocytes; **D**, Heat map of genes related to Th2 CD4^+^ T lymphocytes; **E**, Microarray data of dendritic cells and antigen presentation, apoptosis, Th1 and Th2 CD4^+^ T lymphocytes; **F**, mRNA fold changes of selected genes of dendritic cells and antigen presentation, apoptosis, Th1 and Th2 CD4^+^ T lymphocytes.

**Figure 8:**
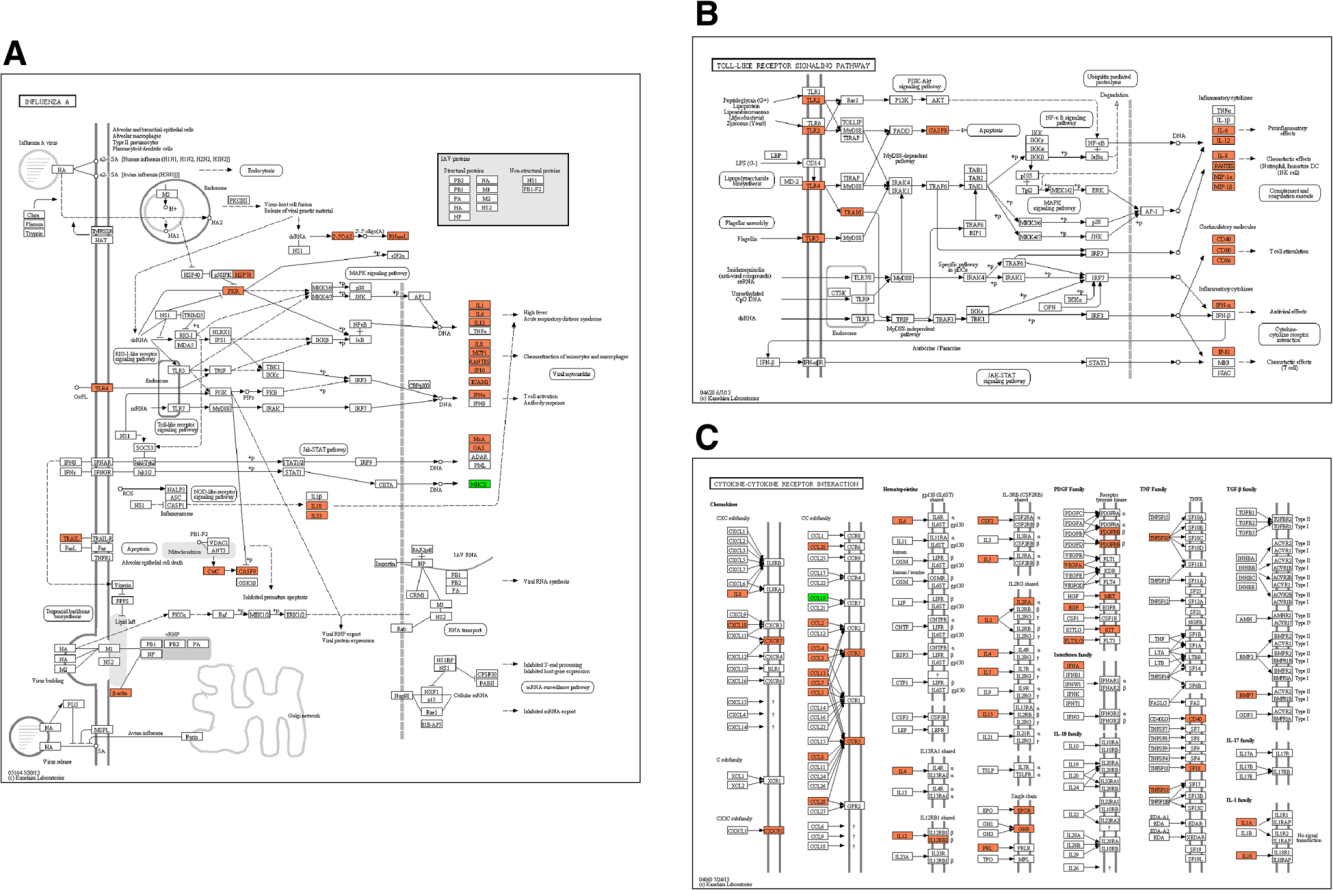
**Ontology of induced genes in the lungs of dogs infected with canine H3N2 influenza virus. A**. Influenza-related gene expression, **B**. Toll-like receptor signaling-related gene expression, **C**. Cytokine-cytokine receptor reaction-related gene expression. Orange box: genes up-regulated in this experiment, Green box: genes down-regulated in this experiment.

We also determined the expression of genes related to adaptive immunity and cell death such as those for dendritic cells, apoptosis, Th1 CD4^+^ lymphocytes, and Th2 CD4^+^ lymphocytes (Figure 7). Transporter 1 (TAP1), which transports various molecules across extra- and intracellular membranes, had a 7596.9-fold increase (Figure 7E) showing the greatest induction among genes related to dendritic cells and antigen presentation. When mRNA of TAP1 was quantified by real-time PCR the induction of it was 6271.1-fold increase (Figure 7F). Genes involved in cell death were also induced in the lungs of the infected dogs. Tumor necrosis factor superfamily 13 (TNFSF13), which induces apoptosis, had a 624.3-fold increase (Figure 7E). When mRNA of TNFSF13 was quantified by real-time PCR, the induction of it was 542.7-fold increase (Figure 7F). NME5 (non-metastatic cells 5), which alters the cellular levels of several antioxidant enzymes, had a 135.3-fold increase (Figure 7E). When mRNA of NME5 was quantified by real-time PCR the induction of it was a 185.7-fold increase (Figure 7F). PYD and CARD domain-containing (PYCARD), which mediates assembly of large signaling complexes in the inflammatory and apoptotic signaling pathways via the activation of caspase, had a 74.3-fold increase and was induced higher than any other apoptosis-related gene (Figure 7E). When mRNA of PYCARD was quantified by real-time PCR the induction of it was a 34.3-fold increase (Figure 7F). Secreted phosphoprotein 1 (SPP1) up-regulating the expression of IFN-γ and IL-12 in Th1 CD4^+^ lymphocytes had a 46.7-fold increase (Figure 7E). When mRNA of SPP1 was quantified by real-time PCR, the induction of it was a 63.8-fold increase (Figure 7F). Chemokine (c-c motif) ligand 7 (CCL7), which attracts monocytes, had the highest induction at a 9.7-fold increase among Th2 CD4^+^ lymphocytes (Figure 7E). When mRNA of CCL7 was quantified by real-time PCR, the induction of it was a 15.3-fold increase (Figure 7F). Ontology of gene expression showed that the infections of dogs with canine H3N2 influenza virus could induce a variety of genes involved in inflammation in lungs of infected dogs (Figure 8).

We think that the immune responses elicited in the dogs infected with canine H3N2 influenza virus may also contribute to the pathogenesis of influenza.

## Discussion

Dogs live in close contact with humans. We characterized the pathogenicity of the avian origin H3N2 influenza virus in dogs. The infected dogs suffered from severe clinical signs, such as high fever, reduced body weight, and pneumonia. The canine H3N2 influenza virus could be efficiently transmitted via aerosols from the infected dogs to naïve dogs. Analysis of transcription induction in the lungs of infected dogs showed that a variety of genes related to innate and adaptive immunity and cell death were up-regulated.

Our data show that dogs infected with a recently isolated canine H3N2 influenza virus suffered from high fever and severe interstitial pneumonia. The previous study using 2007 canine H3N2 influenza virus also showed that the infected dogs suffered from high fever and the interstitial pneumonia and bronchioalveolitis [13].

Our results suggest that a canine H3N2 influenza virus is well adapted in dogs since this virus could efficiently transmit itself through aerosols. The aerosol transmission studies were performed to evaluate the potential transmission of influenza virus among humans using an animal model [30-34]. The transmission efficiency of avian origin H5N1 influenza virus in ferrets has been previously studied [30,31]. The reassortant H5HA/H1N1 virus containing H5 HA with four mutations and the remaining 7 genes from the 2009 pandemic H1N1 influenza virus could transmit among ferrets via droplets, and did not cause severe clinical signs such as death in the transmitted ferret [30]. Another study on the aerosol transmission of avian origin H5N1 influenza virus suggested that the subsequent serial passages of an H5N1 influenza virus in ferrets could result in airborne transmission among ferrets. These transmissible H5N1 viruses contained four amino acid substitutions in the receptor-binding amino acids of HA, and one in the polymerase complex protein basic polymerase 2 [31]. The transmission study in the ferrets carrying avian origin H9N2 influenza virus, which is a potential threat to humans, showed that the H9N2 influenza virus could be transmitted by direct contact with the ferrets, but it could not be transmitted to naïve ferrets via aerosols [32]. The swine-originated, pandemic H1N1 influenza virus of 2009 was easily transmissible among ferrets [33,34]. When the ferrets were infected with the pandemic H1N1 influenza virus or the seasonal H1N1 influenza virus, both viruses were found to be efficiently transmissible among ferrets via aerosols [34]. In addition, the 2009 pandemic H1N1 influenza virus was transmitted among ferrets via contact and respiratory droplet exposure, before the earliest clinical sign of fever was detected [34].

Extensive transcriptional genomic analysis revealed that genes related to innate and adaptive immunity, inflammation, and apoptosis were highly active in the lungs of the dogs infected with canine H3N2 influenza virus, more than the lungs of PBS mock-infected dogs. The resulting immune responses of the dogs may also be responsible for contributing to the pathogenicity of the virus. Previous global transcriptional analysis studies also attributed the immune responses, including inflammation and apoptosis, to the enhanced pathogenicity of influenza viruses in animals [35-37]. A global transcriptional analysis was performed in macaques infected with the 1918 pandemic H1N1 or the avian origin H5N1 influenza virus [35]. Specific groups of genes related to inflammation and cell death were elevated in the bronchial tissues of macaques infected with the 1918 pandemic H1N1 influenza virus, but were downregulated by the avian origin H5N1 influenza virus [35]. When mice were infected with the 1918 pandemic H1N1 influenza virus, the genes associated with pro-inflammatory and cell death pathways were seen to be upregulated 24 h after infection, and remained so until death at 5 days [36]. Extensive transcriptional genomic profiles in the lungs of the ferrets showed that the interferon-response genes were more highly expressed in the avian origin H5N1-infected ferret lungs than in the lungs of the ferrets infected with the H3N2 influenza virus, and strong CXCL10 gene expression was induced in the lungs of the ferrets infected with the avian origin H5N1 influenza virus [37].

Our data show that the gene expression in the lungs of dogs infected with canine H3N2 influenza virus was a little different between data of microarray and qPCR even though the overall patterns are related. The discrepancy between both methods may be due to the inherent pitfall of both methods that may influence the data obtained from each method as described in the previous studies [38-40].

In conclusion, our data suggest that the canine H3N2 influenza virus may be well adapted to infecting dogs, and that the pathogenesis of dogs infected with the canine H3N2 influenza virus may be due to the elevated expression of genes associated with immune responses and cell death in the lungs.

## Competing interests

The authors declare that they have no competing interests.

## Authors’ contributions

YMK and HMK carried out the experiments on clinical observation, measurement of viral titers, tissue staining and microarray, and drafted the manuscript. KBK, EHP, and JY helped with the animal experiments. SHS conceived the study, and participated in its design and coordination. All authors read and approved the final manuscript.
